# A novel pro-inflammatory protein of *Streptococcus suis* 2 induces the Toll-like receptor 2-dependent expression of pro-inflammatory cytokines in RAW 264.7 macrophages via activation of ERK1/2 pathway

**DOI:** 10.3389/fmicb.2015.00178

**Published:** 2015-03-09

**Authors:** Qiang Zhang, Yujie Yang, Shuxian Yan, Jiantao Liu, Zhongmin Xu, Junping Yu, Yajing Song, Anding Zhang, Meilin Jin

**Affiliations:** ^1^Unit of Animal Infectious Diseases, National Key Laboratory of Agricultural Microbiology, College of Veterinary Medicine, Huazhong Agricultural UniversityWuhan, China; ^2^Key Laboratory of Development of Veterinary Diagnostic Products, Ministry of AgricultureWuhan, China

**Keywords:** *Streptococcus suis* 2, inflammation, pro-inflammatory protein, Toll-like receptor 2, ERK1/2 pathway

## Abstract

*Streptococcus suis* 2 is an important swine pathogen and an emergent zoonotic pathogen. Excessive inflammation caused by *S. suis* is responsible for the high levels of early mortality observed in septic shock-like syndrome cases. However, the mechanisms through which *S. suis* 2 (SS2) causes excessive inflammation remain unclear. Thus, this study aimed to identify novel pro-inflammatory mediators that play important roles in the development of therapies against SS2 infection. In this study, the novel pro-inflammatory protein HP0459, which was encoded by the SSUSC84_0459 gene, was discovered. The stimulation of RAW 264.7 macrophages with recombinant HP0459 protein induced the expression of pro-inflammatory cytokines (IL-1β, MCP-1 and TNF-α). Compared with the wild-type (WT) strain, the isogenic knockout of HP0459 in SS2 led to reduced production of pro-inflammatory cytokines in RAW264.7 macrophages and *in vivo*. The pro-inflammatory activity of HP0459 was significantly reduced by an antibody against Toll-like receptor 2 (TLR2) in RAW264.7 macrophages and was lower in TLR2-deficient (TLR2-/-) macrophages than in WT macrophages. Furthermore, specific inhibitors of the extracellular signal-regulated kinase 1/2 (ERK1/2) pathways significantly decreased the HP0459-induced pro-inflammatory cytokine production, and a western blot assay showed that HP0459 stimulation induced the activation of the ERK1/2 pathway. Taken together, our data indicate that HP0459 is a novel pro-inflammatory mediator of SS2 and induces TLR2-dependent pro-inflammatory activity in RAW264.7 macrophages through the ERK1/2 pathway.

## Introduction

*Streptococcus suis* is a major pathogen responsible for important economic losses to the swine industry worldwide, particularly during the past 20 years (Gottschalk et al., 2010). It causes meningitis, septicemia, pneumonia, endocarditis, arthritis, and other diseases in swine (Wei et al., 2009). Among the 33 serotypes based on capsular antigens that have been described, serotype 2 is the most frequently isolated from diseased pigs, particularly in Europe and Asia (Wisselink et al., 2000). Since the first human case was reported in Denmark in Perch et al. (1968), more *S. suis* infections in humans have been documented in several European and Asian countries as well as in North and South America, Australia, and New Zealand (Wertheim et al., 2009; Gottschalk et al., 2010). For a long time, it has been considered to be a fact that *S. suis* only infects people working with pigs or pork-derived products (Arends and Zanen, 1988); however, *S. suis* infections in the general population were recently reported in Southeast and East Asia (Gottschalk et al., 2010). Although most reports concern sporadic cases of infection, a large series of 151 *S. suis* meningitis cases was recently reported in southern Vietnam (Mai et al., 2008). Furthermore, an important outbreak, which involved 215 cases and 38 deaths, occurred in China in during 2005 (Yu et al., 2006). In addition, *S. suis* is considered one of the most important causes of meningitis in humans in various locations, including Vietnam, Thailand, and Hong Kong (Suankratay et al., 2004; Hui et al., 2005; Ip et al., 2007; Mai et al., 2008). These findings emphasize the importance of *S. suis* as an emerging zoonosis and indicate that *S. suis* represents a significant public health concern (Fittipaldi et al., 2012). The increased severity of *S. suis* infection in humans underscores the critical need to better understand the factors associated with the pathogenesis of *S. suis* infection (Gottschalk and Segura, 2000).

Although several virulence-related molecules have been proposed, only the capsular polysaccharide (CPS) has been proven to play a critical role in the virulence of *S. suis* (Charland et al., 1998; Smith et al., 1999; Segura et al., 2004). Some putative virulence factors have also been reported in *S. suis*, such as suilysin, muramidase-released protein (MRP), subtilisin-like protease (SspA), and LTA D-alanylation (DltA; Smith et al., 1992; Lun et al., 2003; Fittipaldi et al., 2008; Bonifait et al., 2011); however, the current knowledge on the pathogenesis of *S. suis* infection remains limited (Baums and Valentin-Weigand, 2009). To cause disease, *S. suis* must breach epithelial barriers, reach and survive in the bloodstream, invade different organs and cause exaggerated inflammation (Fittipaldi et al., 2012). The upregulated expression of several pro-inflammatory cytokines and chemokines, such as tumor necrosis factor-α (TNF-α), interleukin-1β (IL-1β), IL-6 and monocyte chemotactic protein-1 (MCP-1), has been reported in *S. suis* infection (Gottschalk and Segura, 2000; Segura et al., 2002; Al-Numani et al., 2003). Furthermore, inflammation is thought to be responsible for most clinical signs of meningitis, septicemia and sudden death (Segura et al., 2006). *S. suis* sequence type (ST) 7 was recognized as the causative agent for the Sichuan outbreak, and streptococcal toxic shock-like syndrome (STSLS) was observed for the first time in this large outbreak (Ye et al., 2006). A previous study showed that the increased virulence of *S. suis* ST7 is associated with an increased ability to stimulate excessive pro-inflammatory cytokines that may be responsible for the shock syndrome (Zheng et al., 2008). In addition, the most important clinical feature associated with *S. suis* is meningitis in pigs (Gottschalk and Segura, 2000); however, the mechanisms of *S. suis* crossing the blood–brain barrier (BBB) to cause meningitis are poorly understood. Even so, some mechanisms, such as the up-regulation of pro-inflammatory cytokines and increased leukocyte trafficking, have been proposed to contribute to the breakdown of the BBB (Vadeboncoeur et al., 2003; Adam et al., 2004; Jobin et al., 2005; Tenenbaum et al., 2005). The activation of the innate immune response depends on the recognition of pathogen-associated molecular patterns (PAMPs). Toll-like receptors (TLRs) are critical sensors that activate the innate immune response (Beutler, 2009; Kawai and Akira, 2010). For example, TLR2 can form heterodimers with TLR1 or TLR6 to recognize bacterial lipoprotein, lipoteichoic acid (LTA), peptidoglycans (PGNs) and zymosan and induce the release of many cytokines and chemokines responsible for inflammation (Akira and Hemmi, 2003; Beutler, 2004; Lachance et al., 2013). Many previous studies have reported that TLR2 is the major (but not exclusive) immune receptor involved in *S. suis* recognition (Graveline et al., 2007; Li et al., 2010; Lecours et al., 2012).

As mentioned above, inflammation has been thought to be a hallmark of *S. suis* infection (Gottschalk et al., 2007). However, the research on inflammation induced by *S. suis* remains limited. Therefore, it is important to identify novel pro-inflammatory mediators of *S. suis* in order to improve our understanding of the mechanism of inflammation induced by this pathogen. In our previous study, more than 50 extracellular proteins of *S. suis* were expressed, including membrane proteins, secreted proteins and cell wall proteins. And several pro-inflammatory proteins were identified, of which a novel protein HP0459 displayed rather robust pro-inflammatory activity (data not shown). In this study, through measuring the IL-1β and MCP-1 levels by relative quantitative polymerase chain reaction (qPCR) and enzyme-linked immunosorbent assay (ELISA), the pro-inflammatory ability of HP0459 was examined. We investigated the recognition receptor and signal transduction pathway through which HP0459 induces IL-1β, TNF-α and MCP-1 in RAW264.7 macrophages. As a result, we elucidated the mechanism through which HP0459 stimulation induces pro-inflammatory cytokine production.

## Materials and Methods

### Bacterial Strains, Plasmids and Growth Conditions

*Streptococcus suis* serotype 2 strain SC-19, which was isolated from the brain of a dead pig during the epidemic outbreak in Sichuan province of China in 2005, was selected as the wild-type (WT) strain. SC-19 was grown in Tryptic Soy Broth (TSB) or on Tryptic Soy Agar (TSA) plates (Difco, MI, USA) with 5% newborn bovine serum (Sijiqing Biological Engineering Materials Co., Ltd., Hangzhou, China) at 37°C (Li et al., 2013). A temperature-sensitive *S. suis*–*Escherichia coli* shuttle vector (pSET4s) was used to construct the *Δhp0459* mutant, which carries a spectinomycin resistance gene (*spc^r^*). pSET2, a *S. suis–E. coli* shuttle vector carrying *spc^r^*, was used in the construction of the complementary bacterium (Takamatsu et al., 2001).

### Cell Culture

RAW 264.7 macrophages were grown in Dulbecco’s modified Eagle’s medium (DMEM) supplemented with 10% fetal bovine serum in a 5% CO_2_ atmosphere at 37°C (Kang et al., 2009). The primary mouse macrophages were isolated from TLR2-deficient (TLR2-/-; JAX^®^ Mice) and WT mice. The mice were injected intraperitoneally (i.p.) with 4% thioglycolate, and peritoneal exudate cells were harvested 4 days later (van Lint et al., 2010). More than 90% of the exudate cells were identified as macrophages by microscopic analysis and non-specific esterase staining (Sodhi et al., 2005). The macrophages were plated at a density of 10^6^ cells per well in 12-well plates.

### Cloning, Expression, Purification and Endotoxin Removal of HP0459 Protein

The HP0459 protein, which was reported as a secreted protein in a previous study, was encoded by SSUSC84_0459 (Liu et al., 2009). The HP0459 protein was cloned and purified according to published methods (Liu et al., 2012). Briefly, the *hp0459* gene was amplified from the chromosomal DNA of SC-19 by PCR using the primers listed in Table **Table 1**. The purified PCR product was inserted into pET-28a and harbored in *E. coli* BL21 (DE3) cells. HP0459 was induced with 0.5 mM isopropyl-b-D-thiogalactopyranoside (IPTG) and purified by ultrasonication and Ni-NTA agarose chromatography. The endotoxin in the purified recombinant protein was removed using an Endotoxin Removal Kit (Genmed Scientifics Inc. USA), and the endotoxin level was tested using a Quantitative Chromogenic Tachypleus Amebocyte Lysate For Endotoxin Detection kit (Chinese Horseshoe Crab Reagent Manufactory Co., Ltd., Xiamen, China; Liu et al., 2011). The protein was then treated using a 0.22-μm filter. After the above-mentioned treatment, the HP0459 protein was stored at -80°C.

**Table 1:** Oligonucleotide primers used in this study.

Primers	Primers sequence (5′–3′)^a^	Functions
*hp0459*-F	CCCGAATTCACGGAGGTAGCTAACGAAC	For amplification of the *hp0459* ORF gene
*hp0459*-R	CCCCTCGAGTTATTCGGGTGTTGTAAATAG	
*hp0459*-L1	AATGAATTCTTCCTGATAAGAAGGTGGCTAAC	Upstream border of *hp0459*
*hp0459*-L2	AACGTCGACCCTGAAGGATCCTAATGAAGTTT	
*hp0459*-R1	TTAGTCGACGCAATGTAATAACTCGAACTAG	Downstream border of* hp0459*
*hp0459*-R2	CTCAAGCTTGACTAAACCATTAAGCCA	
c*hp0459*-1	CGCGCATGCCCTTTATTATGTCAAGTTCAGAT	To complement *hp0459* in the PCR assays
c*hp0459*-2	CGCGAATTC TTATTCGGGTGTTGTAAATAG	
MCP1-F	TGGGTCCAGACATACATTA	For qPCR assay
MCP1-R	TCAGATTTACGGGTCAACT	
TNFα-F	CGATGAGGTCAATCTGCCCA	For qPCR assay
TNFα-R	CCAGGTCACTGTCCCAGCATC	
IL1β-F	CACCTGGTACATCAGCACCTCAC	For qPCR assay
IL1β-R	CATCAGAAACAGTCCAGCCCATAC	
GAPDH-F	CGTCGGTGCTGAGTATGTCGT	For qPCR assay
GAPDH-R	CAGTCTTCTGGGTGGCAGTGAT	
P1	TAGTTTCTGATAAACTTCATTAGGA	To identify the *hp0459* gene by PCR
P2	AAATGCGCTCGAAATGA	
P3	TGGAAATGTTCAAGTCAACC	To identify the *gdh* gene by PCR
P4	CGTTTTTCTTTGATGTCCAC	
P5	GCACAGATGCGTAAGGAG	To identify the pSET4s by PCR
P6	ACTCTGTAGCACCGCCTA	


^a^The underlined nucleotides denote enzyme restriction sites.

### RNA Extraction and qPCR Assay

After the treatment of RAW 264.7 cells with HP0459 at 10 μg ml^-1^ for 10 h, the total RNA of the cells was extracted with the TRIzol^®^ reagent (Invitrogen, Paisley, UK), according to the manufacturer’s guidelines. The RNA pellets were suspended in RNase-Free water, and the DNA contamination of the RNA was removed by DNase treatment (Promega, Madison, WI, USA). cDNA was obtained from 4 μg of RNA by reverse transcription using AMV reverse transcriptase (TAKARA, Japan) and the oligo-dT primer (300 pmol) in a total reaction volume of 40 μl (Moore et al., 2005). Relative quantitative PCR (qPCR) was performed to measure the mRNA levels of pro-inflammatory cytokines (IL-1β, MCP-1 and TNF-α) using a SYBR green PCR Kit (Roche) and the ABI ViiA7 instrument. Glyceraldehyde-3-phosphate dehydrogenase (GAPDH) was used as the reference gene, and all of the primers used in the qPCR assay are listed in Table **Table 1**. The data were analyzed using the ViiA7 software (Applied Biosystems; Zhao et al., 2014).

### Enzyme-Linked Immunosorbent Assay for Cytokines

After treatment of RAW 264.7 cells with HP0459 at 10 μg ml^-1^ for 10 h, the protein levels of IL-1β, MCP-1 and TNF-α in the cell culture supernatants were determined using commercially available ELISA kits (Biolegend) according to the manufacturer’s instructions.

### Generation of an Isogenic *hp0459* Deletion Mutant and Complemented Strains

The construction of the Δ*hp0459*-knockout mutant was performed using a previously described procedure (Takamatsu et al., 2001). Briefly, DNA fragments were amplified from the genomic DNA of SC-19 by PCR using two pairs of specific primers, namely *hp0459*-L1/*hp0459*-L2 and *hp0459*-R1/*hp0459*-R2 (Table **Table 1**), which carry *EcoRI/SalI* and *SalI/HindI* restriction enzyme sites, respectively. The fragments were digested with the corresponding restriction enzymes and sequentially ligated into the temperature-sensitive *S. suis–E. coli* shuttle vector pSET4s to generate the *hp0459*-knockout vector pSET4s*Δhp0459.* To obtain the isogenic mutant *Δhp0459,* competent cells of SC-19 were subjected to electrotransformation with pSET4s*Δhp0459* as described previously (Takamatsu et al., 2001). The suspected mutant was verified by PCR using three pairs of primers: P1/P2 (to identify *hp0459*), P3/P4 (to identify *gdh*) and P5/P6 (to identify the pSET4s).

The complemented strain of *hp0459* was constructed as described previously (Zhang et al., 2012). Briefly, a DNA fragment that contained the *hp0459* gene and its predicted upstream promoter was amplified by PCR using the primers c*hp0459*-1/c*hp0459*-2 (Table **Table 1**), which carry *SphI/EcoRI* restriction enzyme sites, respectively. To generate the recombinant plasmid pSET2C*hp0459,* the fragment was digested with the appropriate restriction enzymes and was cloned into the *E. coli–S. suis* shuttle vector pSET2 carrying the same cohesive terminus. The plasmid was then electrotransformed into *Δhp0459* to obtain the complemented *CΔhp0459* strain.

### Experimental Infections *In Vitro* and *In Vivo*

*In vitro*, RAW 264.7 cells were infected with 5 × 10^6^ CFU of the WT (SC-19), *Δhp0459* or *CΔhp0459* strains in the logarithmic phase of growth. The supernatants were collected for western blot analysis after 10 h at 37°C.

All of the animal studies were performed according to the experimental protocols approved by the Laboratory Animal Monitoring Committee of Hubei Province, China. A total of 125 6-weeks-old female C57BL/6 mice were randomly divided into three groups with 40 mice per group, and the remaining five mice were used as controls. The three groups of mice were challenged intraperitoneally (i.p.) with 5 × 10^8^ CFU log-phase WT (SC-19), *Δhp0459* or *CΔhp0459* strains, respectively. At certain times post infection (3, 6, 9, and 12 h), an equal number of mice in each group were killed. Bacteriological isolation from the blood or part of the spleen was performed essentially as described previously (Zhang et al., 2011), and the lungs or another part of the spleen were used to determine the IL-1β and TNF-α levels by qPCR.

### Investigating the Recognition Receptor of HP0459

Antibody blocking assays were performed to investigate the recognition receptor of HP0459 in RAW264.7 cells using the anti-TLR2 (eBioscience) and anti-TLR4 (BioLegend) antibodies. Briefly, RAW264.7 cells were pretreated using 8 μg of anti-TLR2 and anti-TLR4 antibody for 30 min respectively and then incubated with 10 μg ml^-1^ HP0459 for 10 h. The expression levels of various cytokines were determined by ELISA. According to the conditions of cytokine activation, the recognition receptor of the HP0459 was analyzed. In addition, TLR2-/- macrophages were isolated from TLR2-/- mice to verify the results of the blocking assays.

### Analysis of HP0459-Induced Cell Signal Transduction Pathways

RAW264.7 macrophages (1 × 10^6^ cells ml^-1^) were seeded into 12-well tissue culture plates. The cells were pretreated with the following specific inhibitors for 30 min prior to the addition of HP0459 (Liu et al., 2008): U0126 (for ERK1/2; 10 μM), SP600125 (for JNK; 10 μM), pyrrolidine dithiocarbamate (PDTC; for NF-_κ_B; 20 μM) and LY294002 (for PI3K; 20 μM). All inhibitors were purchased from Cayman Chemical. Culture supernatants were collected at the indicated times and stored at -80°C until assayed.

### SDS-PAGE and Western Blot Analysis

To confirm the HP0459-induced phosphorylation of signal transduction molecules, a western blot analysis was performed. To extract the cytosolic protein, after stimulation with HP0459 (10 μg ml^-1^) for 10 h, RAW264.7 cells were washed with cold PBS and harvested by centrifugation. The pellets were then suspended in RIPA lysis buffer with phosphatase inhibitor (Roche) for 15 min on ice. The protein concentrations in the lysates were quantified with the Bradford protein assay, and 40 μg of proteins were subjected to 12% SDS-PAGE and transferred onto a 0.22-μm nitrocellulose membrane. Subsequently, these proteins were probed with specific Abs against the phosphorylated forms of ERK1/2 and NF-_κ_B p65 (Cell Signaling Technology, Beverly, MA, USA), and β-actin was assessed as an internal control using anti-β-actin antibody (Wuhan PMK Biotechnology Co., Ltd.). The detection of the bands was performed using HRP-conjugated secondary antibody and an enhanced chemiluminescence (ECL) system (Amersham Life Science, Arlington Heights, IL, USA).

### Statistical Analysis

The statistical significance of the data was determined using Student’s *t* test with GraphPad Prism software (San Diego, CA, USA), and all the assays were repeated at least three times. For all tests, a value of *P* < 0.05 was considered as the threshold for significance.

## Results

### Cytokine Secretion from RAW264.7 Cells Stimulated with HP0459

**FIGURE 1:**
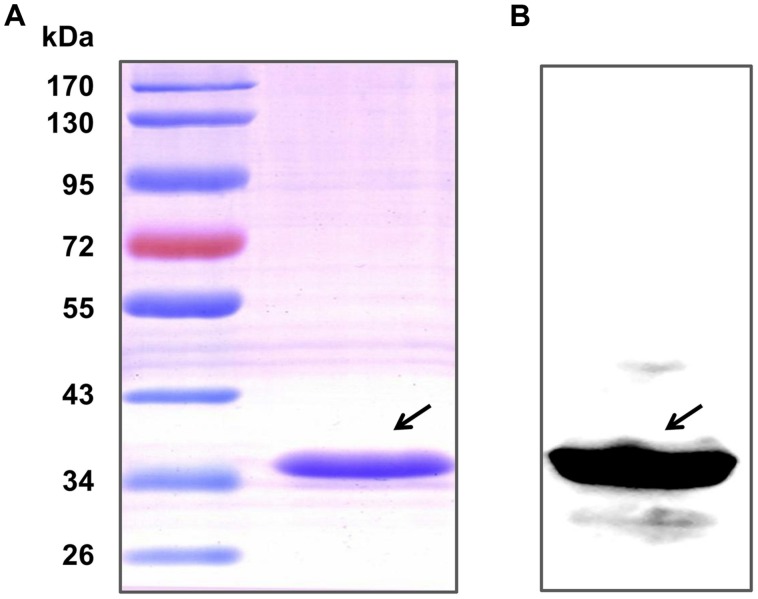
Purification of the recombinant Hp0459 protein. **(A)** SDS-PAGE. **(B)** Western blot analysis. The blot was probed with his tag monoclonal antibody (Cali-Bio).

**FIGURE 2:**
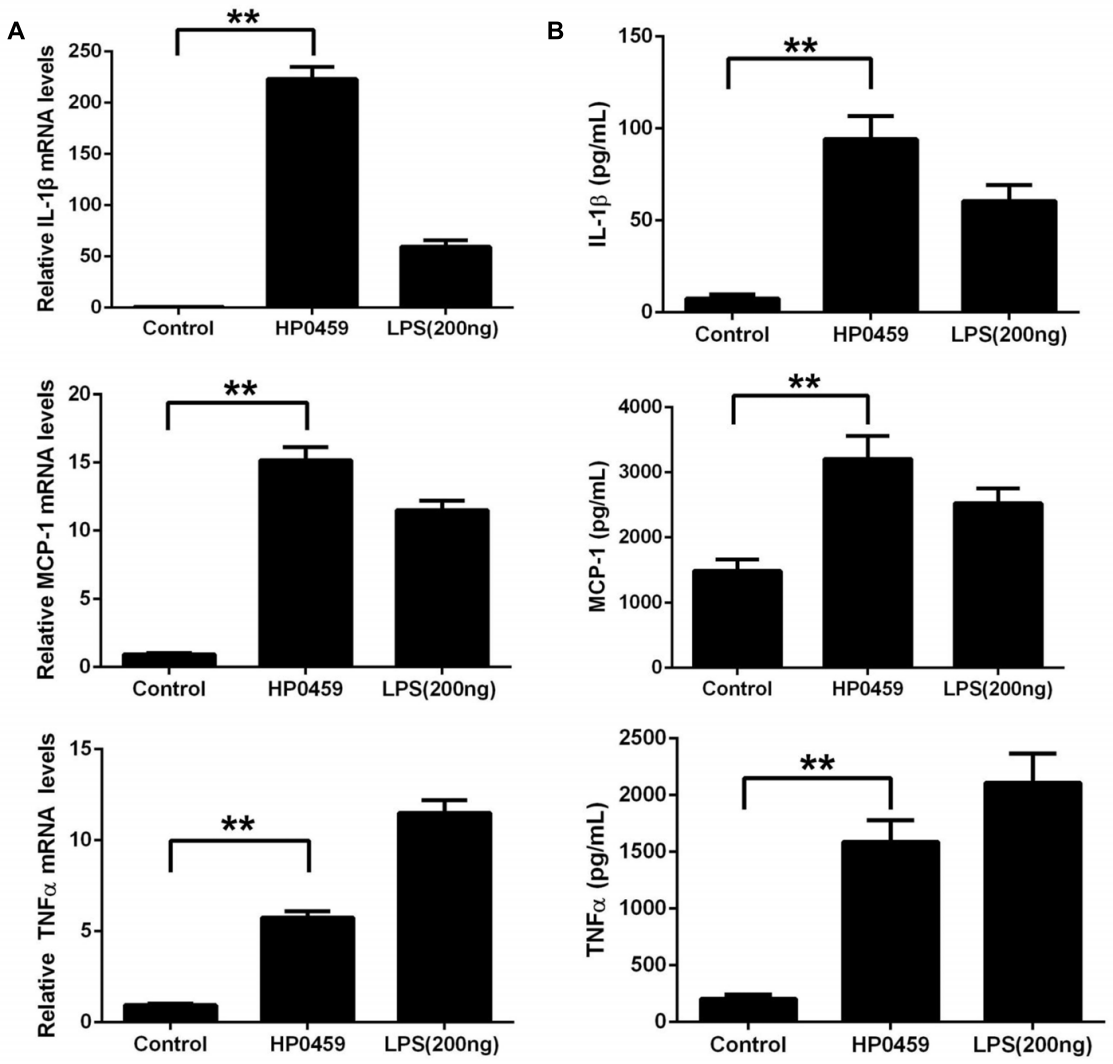
Induction of cytokine mRNA and protein in RAW 264.7 macrophages by recombinant HP0459 stimulation. RAW 264.7 macrophages were incubated with 10 μg ml^-1^ HP0459 and 200 ng ml^-1^ LPS (positive control) for 10 h, as well as single culture media (negative control), **(A)** the cytokine mRNA levels were then determined by qPCR, **(B)** and the protein levels of IL-1β, MCP-1 and TNF-α in the culture supernatants were determined by ELISA. The bars represent the standard errors of the means, based on three independent experiments. **P <0.01.

After purification by Ni-NTA agarose chromatography, the SDS-PAGE (**Figure 1A**) and western blot analysis (**Figure 1B**) of HP0459 revealed that the HP0459 protein was successfully purified. The average endotoxin level in HP0459 was ∼0.05 endotoxin units per milliliter via endotoxin removal. After the above-mentioned treatment, to determine the pro-inflammatory role of HP0459, RAW264.7 cells were stimulated with HP0459 at a concentration of 10 μg ml^-1^ for 10 h and analyzed by qPCR and ELISA respectively. As shown in **Figure 2**, it was confirmed that HP0459 stimulation significantly increased the expression levels of IL-1β, MCP-1, and TNF-α (*P <*0.01) by qPCR (**Figure 2A** and ELISA (**Figure 2B**) analyses.

### Construction and Characterization of the Mutant Strain *Δhp0459*

**FIGURE 3:**
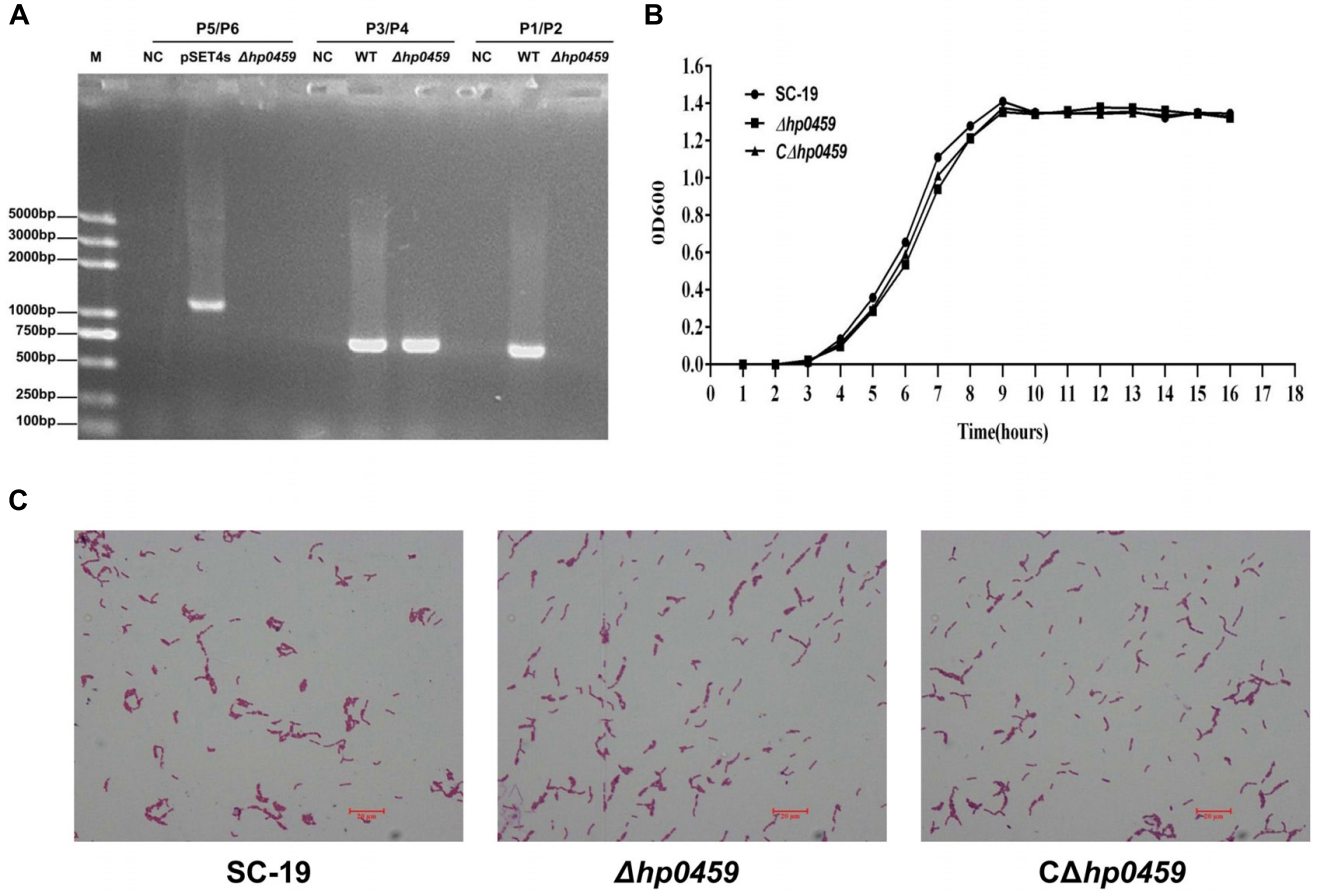
Construction and confirmation of *Δhp0459* and the complementary strain *CΔhp0459*. **(A)** Confirmation of the *Δhp0459* mutant by PCR using the primers pairs P1/P2 (to identify *hp0459*), P3/P4 (to identify *gdh*), and P5/P6 (to identify the pSET4s). **(B)** Growth curves of the SC-19, *Δhp0459* and *CΔhp0459* strains. The bacteria were cultured in TSB containing 5% newborn bovine serum at 37°C. The absorbance at 600 nm was measured at intervals of 1 h. Results shown are representative of three independent experiments. **(C)** Light microscope morphology of the SC-19, *Δhp0459* and *CΔhp0459* strains using Gram staining. The bar indicates the magnification size.

To study the role of HP0459 in the *S. suis*-induced pro-inflammatory response, the HP0459-knockout mutant *Δhp0459* was constructed by homologous recombination, and the double-crossover event was confirmed by PCR (**Figure 3A**). To examine the growth characteristics of the mutants *in vitro,* the OD600 values of cultures of the SC-19, *Δhp0459* and *CΔhp0459* strains in TSB containing 10% newborn bovine serum at 37°C were determined (**Figure 3B**), and Gram-staining of these three strains was also performed (**Figure 3C**). No significant difference in growth was found between the SC-19, *Δhp0459* and *CΔhp0459* strains.

### Pro-inflammatory Attenuation Induced by the *Δhp0459* Strain *In Vitro* and *In Vivo*

**FIGURE 4:**
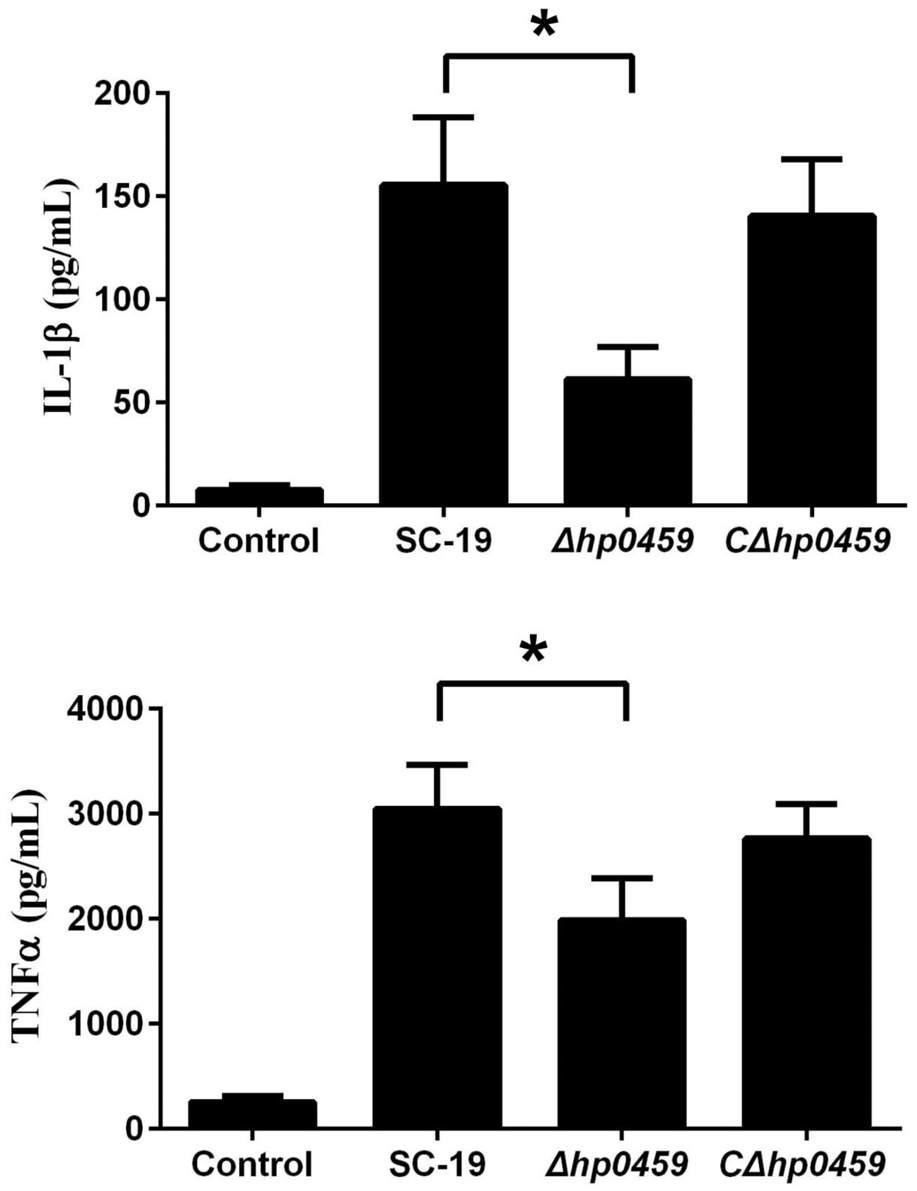
Induction of IL-1βandTNF-α by SS2 strains in RAW 264.7 macrophages. RAW 264.7 macrophages were incubated with the SC-19, *Δhp0459* and *CΔhp0459* strains for 10 h. The levels of IL-1β and TNF-α in the supernatants were measured by ELISA. The bars represent the SEs of the means, based on three independent experiments. *P <0.05.

After construction of the *Δhp0459* strain, the role of HP0459 in the *S. suis*-induced pro-inflammatory response was assessed in RAW264.7 cells *in vitro.* The culture supernatants of RAW264.7 cells incubated with the SC-19, *Δhp0459* and *CΔhp0459* strains were harvested, and the levels of IL-1β and TNF-α in these supernatants were measured by ELISA. The results showed that the pro-inflammatory activity of the *Δhp0459* strain was significantly lower than those of the SC-19 and *CΔhp0459* strains (**Figure 4**). This finding suggested that HP0459 plays an important role in the *S. suis*-induced pro-inflammatory response *in vitro*.

**FIGURE 5:**
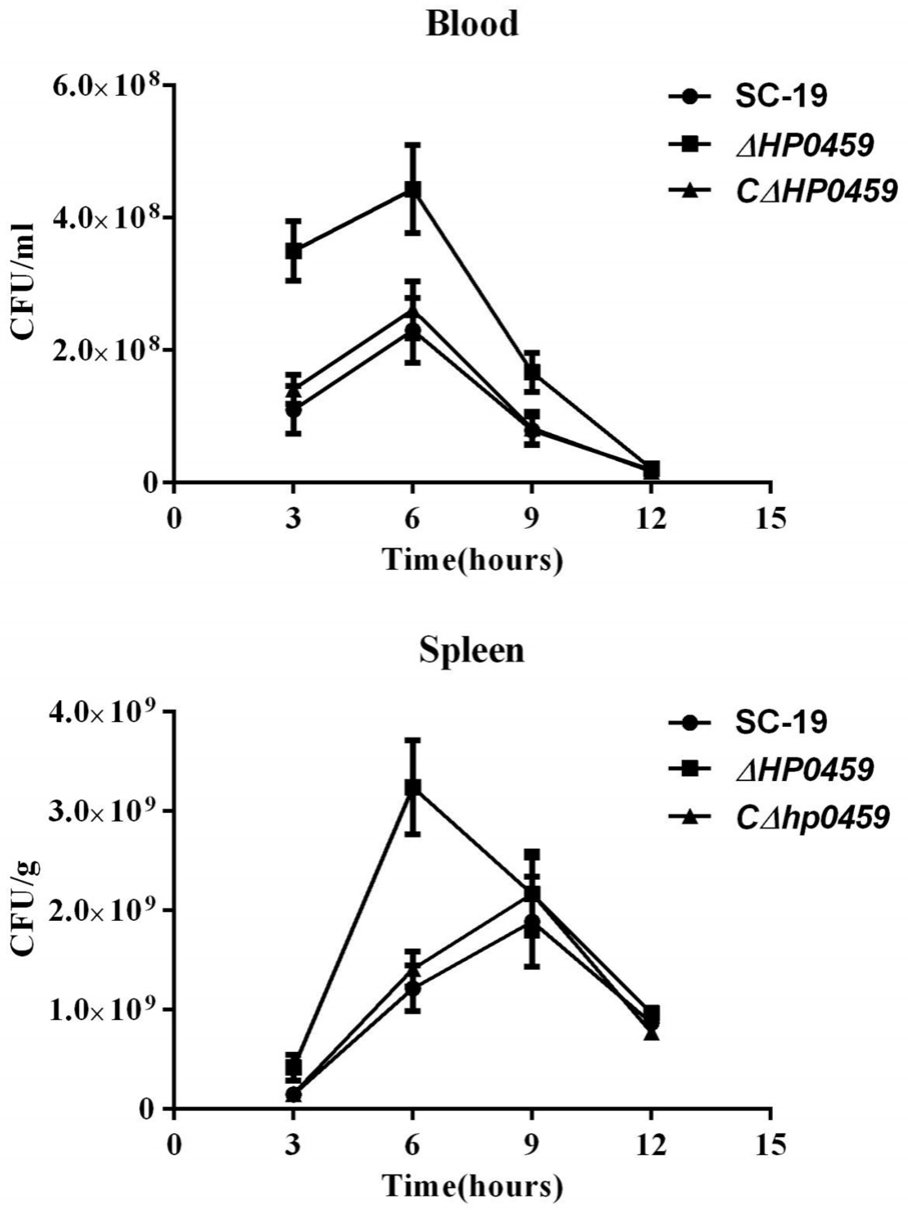
Bacteriological isolation from blood and spleen. C57BL/6 mice were challenged with 5 × 10^8^ CFU of the log-phase SC-19, *Δhp0459* or *CΔhp0459* strains. After infection for 3, 6, 9, and 12 h, bacteriological isolation from the blood and spleens was performed. Results shown are representative of three independent experiments.

**FIGURE 6:**
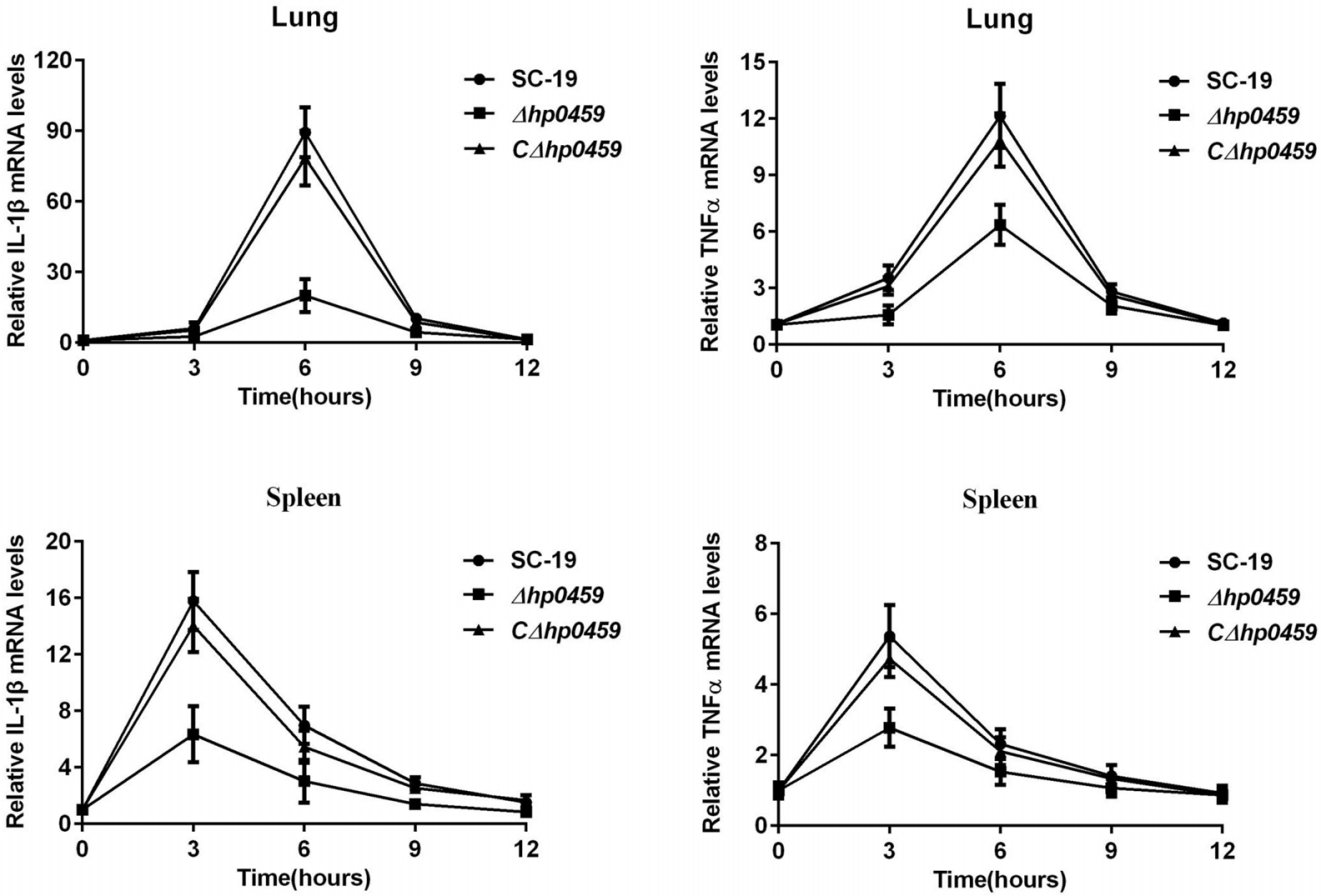
Induction of IL-1βandTNF-α mRNA *in vivo* by stimulation with SS2 strains. C57BL/6 mice were challenged with 5 × 10^8^ CFU of the log-phase SC-19, *Δhp0459* or *CΔhp0459* strains. After infection for 3, 6, 9, and 12 h, the mRNA levels of IL-1β and TNF-α in the lungs and spleens were measured by qPCR. Results shown are representative of three independent experiments.

The role of HP0459 *in vivo* in the *S. suis*-induced pro-inflammatory response was then assessed using an experimental infection model in C57BL/6 mice. Bacteriological isolation from the blood and part of the spleen showed that, compared with the SC-19 and *CΔhp0459* strains, the bacterial content of the *Δhp0459* strain was significantly increased (**Figure 5**). At the same time, the lungs and another part of the spleen from infected and control mice were separated to extract the total RNA, and the mRNA levels of IL-1β and TNF-α were measured by qPCR. The results indicated that the mRNA levels of IL-1β and TNF-α induced by the *Δhp0459* strain *in vivo* were significantly lower than those induced by the SC-19 and *CΔhp0459* strains (**Figure 6**). Thus, the HP0459 protein plays an important role in the *S. suis*-induced pro-inflammatory response.

### HP0459 Protein Induced the Expression of IL-1β, MCP-1 and TNF-α by TLR2

**FIGURE 7:**
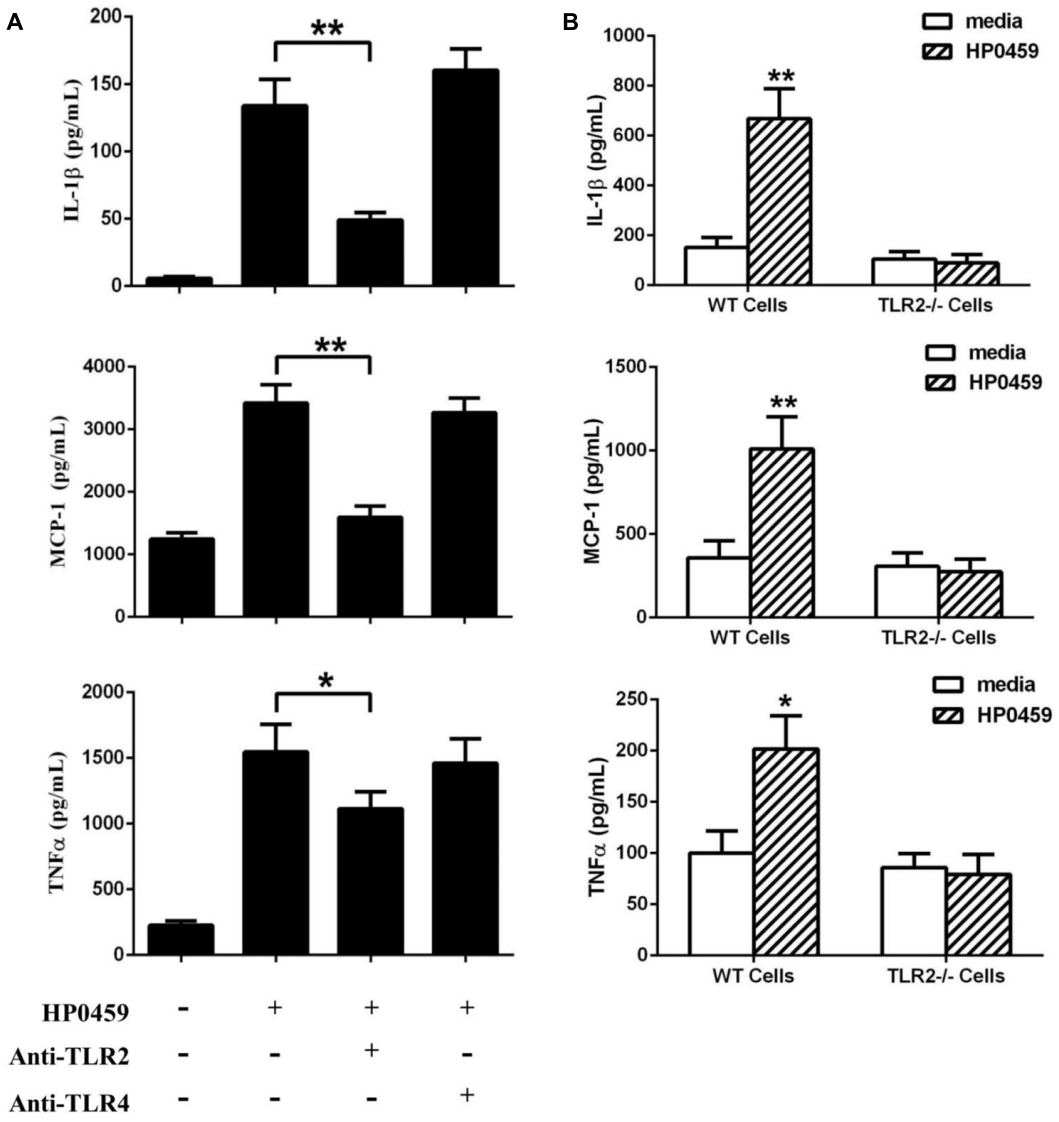
Recognition receptor of the HP0459-stimulated pro-infla-mmatory response. **(A)** Antibody blocking assays. After pretreatment with 8 μg of anti-TLR2 and anti-TLR4 antibody for 30 min, RAW 264.7 macrophages were incubated with 10 μg ml^-1^ HP0459 for 10 h. The expression levels of various cytokines were then determined by ELISA. **(B)** Primary peritoneal macrophages were isolated from TLR2-/- and WT mice and incubated with 10 μg ml^-1^ HP0459 for 10 h. The induction of IL-1β, MCP-1 and TNF-α in the supernatants was then determined by ELISA. The bars represent the SEs of the means, based on three independent experiments. *P <0.05, **P <0.01.

To determine the recognition receptor responsible for the HP0459-mediated induction of cytokines, antibody blocking assays were performed. Compared with the positive control, anti-TLR2 antibody could significantly reduce the expression of IL-1β, MCP-1 and TNF-α induced by HP0459, whereas anti-TLR4 antibody could not (**Figure 7A**). The results showed that the HP0459-induced cytokine secretion may depend on TLR2. To verify this result, TLR2-/- and WT macrophages were isolated from TLR2-/- and WT mice, respectively, and were incubated with HP0459. The results show that HP0459 could significantly induce cytokine secretion in WT macrophages but not in TLR2-/- cells (**Figure 7B**), demonstrating that the HP0459 protein can induce a TLR2-dependent pro-inflammatory response.

### P0459-Induced Cell Signal Transduction Pathways in RAW264.7 Cells

**FIGURE 8:**
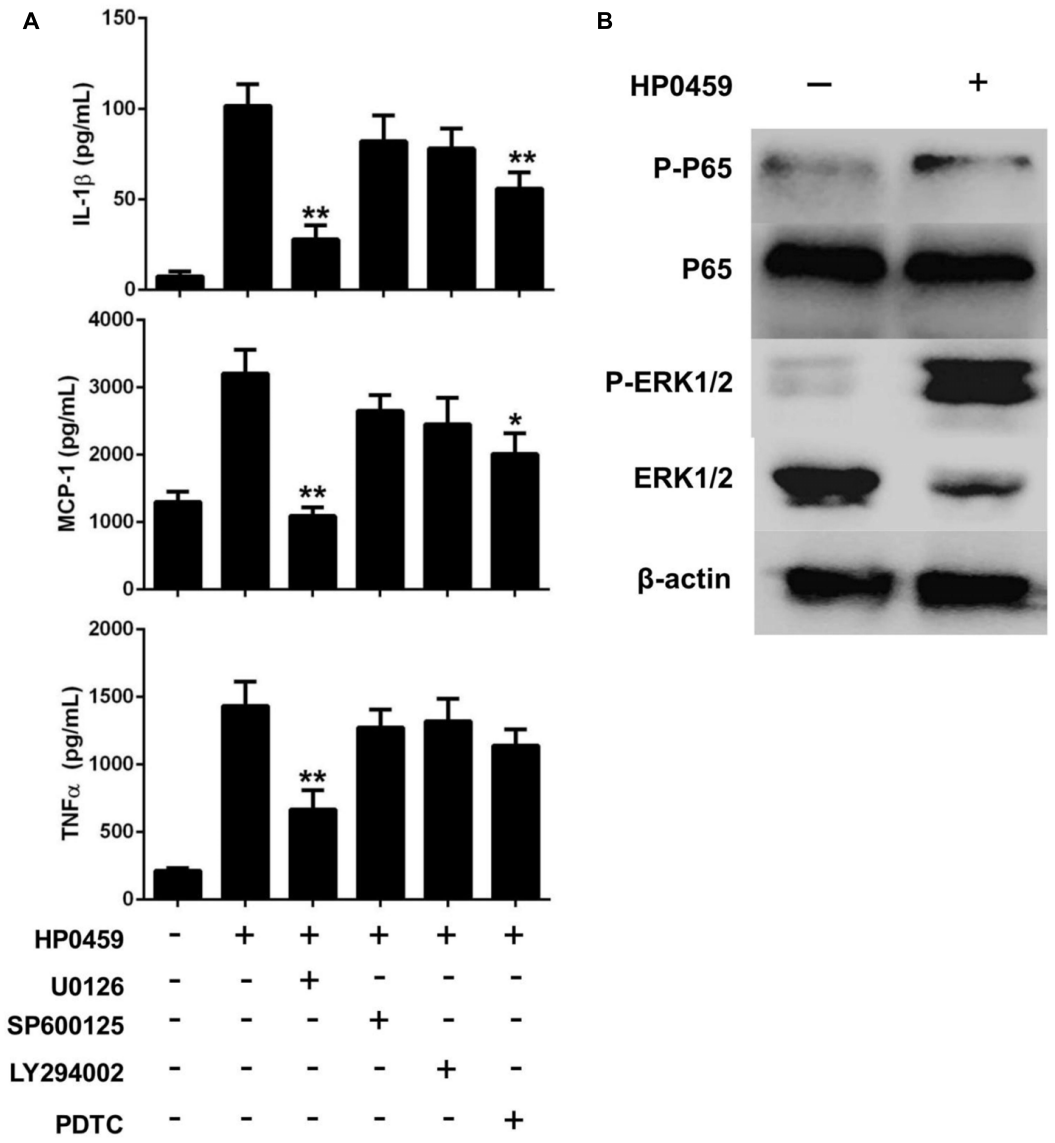
Signal transduction pathways of the HP0459-stimulated pro-inflammatory response in RAW 264.7 macrophages. **(A)** Effect of ERK 1/2 (U0126), JNK (SP600125), PI3K (LY294002) and NF-κB (PDTC) inhibitors on cytokine production during HP0459 stimulation. After incubation with the inhibitors for 30 min, RAW 264.7 macrophages were stimulated with 10 μg ml^-1^ HP0459 for 10 h. The cytokine levels were then determined by ELISA. Data are expressed as the mean ± SD of three independent experiments. **(B)** HP0459-induced phosphorylation of ERK 1/2 MAPK in RAW264.7 macrophages. RAW264.7 macrophages were stimulated with HP0459 (10 μg ml^-1^) for 10 h. The cell lysates were analyzed by western blotting using specific antibodies against ERK 1/2 MAPK, phospho-ERK 1/2 MAPK, NF-κB and phospho-NF-κB. At the same time, β-actin was assessed as an internal control using anti-β-actin antibody. Results shown are representative of three independent experiments. *P <0.05, **P <0.01.

Next, to further elucidate the mechanisms through which the HP0459 protein induced cytokine secretion, we investigated the cell signal transduction pathways in HP0459-stimulated RAW264.7 cells. RAW264.7 cells were pretreated with specific inhibitors of several cell signal pathways for 30 min and were then were incubated with HP0459 for 10 h. The levels of IL-1β, MCP-1, and TNF-α in the supernatants were quantified by ELISA. As shown in **Figure 8A**, the ERK 1/2 MAPK inhibitor (U0126) significantly decreased the HP0459-induced cytokine production, and the NF-_κ_B inhibitor (PDTC) induced a lower degree of reduction. This result suggested that the HP0459-induced cytokine production likely primarily depends on the phosphorylation of ERK 1/2 MAPK. To verify this hypothesis, we performed a western blotting analysis to measure the phosphorylation of ERK 1/2 MAPK and NF-_κ_B in RAW264.7 cells induced by HP0459 stimulation (**Figure 8B**). The results showed that the phosphorylation of ERK 1/2 MAPK was significantly enhanced, whereas the phosphorylation of NF-_κ_B was slight. In addition, a western blot analysis with anti-actin antibody was used to confirm that equivalent amounts of the samples were loaded into the gels. These results suggested that signal transduction pathway ERK 1/2 MAPK play a primary role in the pro-inflammatory response induced by HP0459 stimulation in RAW264.7 cells.

## Discussion

Due to the high prevalence of *S. suis*-induced diseases in humans in Southeast and East Asia, pathogen has been increasingly investigated (Fittipaldi et al., 2012). Although the pathogenesis of* S. suis* infection is not entirely known at present, several viewpoints have been generally recognized, and one of these is that inflammation plays an important role in *S. suis* infection (Segura et al., 2006). After pathogen invasion into a host, the innate immune system of the host will recognize key molecular signatures borne by PAMPs to activate the inflammatory response in order to clear pathogens (Martinon and Tschopp, 2005). However, during *S. suis* infection, the inflammatory response is excessively activated and thus plays an important role in most clinical signs of *S. suis* disease, including meningitis, septicemia and sudden death (Dominguez-Punaro Mde et al., 2008). Thus, it is significant to identify the pro-inflammatory molecules of *S. suis* in order to understand its pathogenesis. In this study, it was found that HP0459 could significantly activate inflammatory response in RAW264.7 cells, but heat-killed HP0459 couldn’t (data not shown). Further, we elucidated the mechanism of HP0459 inducing inflammation. This result contributes to the understanding of the excessive inflammation induced by *S. suis.*

During *S. suis* infection, this pathogen can be recognized by some receptors, including CD14, TLR2, TLR6, and TLR9. CD14 has been considered one of the recognition receptors of LPS, the major component of the outer membrane of Gram-negative bacteria (Wright et al., 1990), and CD14 has been shown to be important in the recognition of cell wall constituents of Gram-positive bacteria (Wright, 1995; Moreillon and Majcherczyk, 2003). A previous study proved that *S. suis* can mediate CD14-dependent cytokine and chemokine production by human monocytes (Segura et al., 2002). Because CD14 lacks transmembrane and intracellular domains, it is not able to transduce the signal by itself. Thus, to activate the CD14-dependent inflammatory response, additional transmembrane receptors, such as TLR2, are required (Manukyan et al., 2005). TLR2, as a major pattern recognition receptor for ligands derived from Gram-positive bacteria (Kawai and Akira, 2005), has been shown to play an important role during *S. suis* ST1 strain interactions with mouse, swine and human cells (Graveline et al., 2007; Zheng et al., 2011, 2012). In addition, it has been indicated that not only TLR2 but also TLR6 and TLR9 play an important role on cell activation through *in vitro* studies carried out with the whole cells of the epidemic ST7 strain and human peripheral blood cells (Zheng et al., 2012). In the present study, using antibody blocking and TLR2-/- mouse macrophages assays, we found that TLR2 plays an important role in the inflammatory response activated by HP0459. This is consistent with *S. suis*-induced pro-inflammatory response, which is primary TLR2-dependent. However, LipoP prediction of HP0459 displayed that the signal peptide of HP0459 belonged to SpI (lipoprotein signal peptide belonged to SpII; Hutchings et al., 2009). This result suggested that the recognition of HP0459 may be different from bacterial lipoproteins. So, the mechanisms of HP0459 recognized by TLR2 need further research. We have proved that HP0459 contributes to pro-inflammatory response during *S. suis* infection. And it is known that there is a close link between excessive inflammation and the development of *Streptococcus* toxic shock syndrome (STSS; Zhao et al., 2011). This implies that HP0459 may be responsible for the pathogenesis of STSS caused by *S. suis* 2.

Compared with the SC-19 strains, the pro-inflammatory activation of the *Δhp0459* strain *in vivo* was found to be significantly reduced in the present study. However, the mouse experiment showed that the lethality of the SC-19 and *Δhp0459* strains were not significant different after challenge with the same CFUs (data not shown). To explain this result, we performed bacteriological isolation from the blood and spleen, and compared with the SC-19 and *CΔhp0459* strains, the bacteria content of the *Δhp0459* strain was significantly increased (**Figure 5**). It is known that higher bacterial counts of pathogenic bacteria* in vivo* may be an important cause leading to disease worsening (Sullivan et al., 1982). Thus, this finding suggests that inflammation is mainly but not exclusively responsible for the pathology of *S. suis* and that the bacteria content also plays an important role.

Finally, our data identified a novel pro-inflammatory protein denoted HP0459 from *S. suis*. Further, we demonstrated that HP0459 induces a TLR2-dependent pro-inflammatory response in RAW 264.7 macrophages via activation of the ERK1/2 pathway. These findings could be important for improving our understanding of the excessive inflammation induced by *S. suis* and may aid the development of therapies against SS2 infection.

## Conflict of Interest Statement

The authors declare that the research was conducted in the absence of any commercial or financial relationships that could be construed as a potential conflict of interest.
